# Breba Fruits Characterization from Four Varieties (*Ficus carica* L.) with Important Commercial Interest in Spain

**DOI:** 10.3390/foods10123138

**Published:** 2021-12-17

**Authors:** Dámaris Núñez-Gómez, Pilar Legua, Juan José Martínez-Nicolás, Pablo Melgarejo

**Affiliations:** Centro de Investigación e Innovación Agroalimentario y Agroambiental (CIAGRO), Miguel Hernández University (UMH), Ctra. Beniel kim 3.2., 03312 Orihuela, Spain; dnunez@umh.es (D.N.-G.); p.legua@umh.es (P.L.); juanjose.martinez@umh.es (J.J.M.-N.)

**Keywords:** *Ficus carica* L., breba fruit, fruit quality, variety diversity, nuclear magnetic resonance

## Abstract

Although most of the published articles generalize with the fruit of the fig tree (*Ficus carica* L.), the differentiation between fig and breba is increasingly common in the bibliography. In this regard, keep in mind that the fig tree generally produces two crops a year, the parthenocarpic breba, also called as early fig, and the main non-parthenocarpic crop, the fig proper. In this study, four brebas varieties (‘Colar’, ‘SuperFig1’, ‘Cuello de Dama Negro’ and ‘San Antonio’) were selected in order to identify compositional, nutritional, and chemical diversity. These varieties were chosen for their commercial relevance in Spain. Color (internal and external), fruit and peel weight, size, pH, total soluble solids (TSS), titratable acidity (TA), maturity index (MI), sugar, and organic content were determined for all the breba fruits samples. In addition, polyphenolic profile, amino acids, and volatile aromatic compounds were also identified. The varieties ‘Colar’ and ‘SuperFig1’ showed the highest fruit weight and size, while ‘Cuello de Dama Negro’ presented the higher pulp yield. The higher organic acid and sugar contents were determined for ‘SuperFig1’ and ‘Cuello de Dama Negro’, respectively. Although in low concentrations, the phenolic compound quercetin 3-(6-O-acetyl-beta-glucoside) and the amino acid tyrosine were only detected in the ‘’Cuello de Dama Negra’ and ‘SuperFig1’ fruits, respectively. Of the eighty volatile aromatic compounds identified, only eight were common in four varieties. An important knowledge gap was identified in relation to the characterization of the two *Ficus carica* L. crops, that is, the differentiation and specification in the literature when working with brebas and/or figs.

## 1. Introduction

The fig tree (*Ficus carica* L.), considered one of the oldest fruit trees cultivated in the Mediterranean region, continues to present an important economic and nutritional role today due to the high consumption around the world of its fruit, the fig and breba (early fig) [1,2,3].

Although its origin is indicated in Asia Minor, its cultivation is widespread throughout the world, mainly in the Mediterranean region due to its mild winters and hot and dry summers [4]. Among the Mediterranean producing countries, Turkey, Algeria, Greece, Italy, and Spain stand out, from which about 90% of fig production is obtained [5,6]. In Europe, Spain is the major producer of figs, with around 56,600 tonnes in 2019, representing 43.6% of European production and 4% of the world’s production [7].

In general, and due to their maturity date and bloom, the *Ficus carica* L. fruits, are called brebas and/or figs. The figs appear from the bloom of the year, while the brebas, in those varieties that have this aptitude, are dormant figs that do not begin their development until the following spring. The “biferous” varieties are those that produce a crop of brebas and another of figs, while the “uniferous” produce a single crop of figs. The breba are larger and juicier and are normally eaten fresh and figs are smaller and can be eaten both fresh and dry [5,8].

Early fig fruits or brebas are popular for their sweet, smooth taste, and fluffy, fresh texture. Its consumption is mainly fresh and dry but also processed as a jam. On a nutritional level, breba fruits are a valuable source of minerals, vitamins, sugars, antioxidants, and phenolic compounds among other important metabolites. Due to its nutraceutical characteristics, records have been found that indicate its use since ancient times by different systems of traditional medicine, such as Siddha, Unani, and Ayurveda, to treat different cardiovascular, gastrointestinal, and respiratory diseases [2,4].

However, it should be noted that, although the fig tree (*Ficus carica* L.) is the most important species at a commercial level, it consists of more than 600 varieties with significant genetic diversity and verified by numerous studies [3,9,10,11,12]. In this sense, and given that the varietal variation has a direct influence on the physicochemical and pomological characteristics of the fruits, the objective and specific characterization of each of the commercial varieties of the fig tree is necessary.

Thus, the main objective of this work was the pomological and physicochemical characterization of the breba fruit of four commercial varieties, the varieties ‘Colar’, ‘SuperFig1’, ‘Cuello de Dama Negro’ and ‘San Antonio’. The four breba varieties were chosen both for their availability and coexistence in the same commercial farm (guaranteeing homogeneous soil, climatic and crop management conditions), both for their national and international importance, that is to say, their commercial representation mainly destined for fresh consumption [5,8,13]. Most of the varieties cultivated in Spain, as the varieties studied in the present work, belong to the parthenocarpic and biferous group but there are also those of the San Pedro type (i.e., ‘Nazaret’ and ‘Tiberio’ varieties) mainly cultivated in Spanish western. In other countries, such as Turkey, Tunisia, Greece, part of Portugal, and California, the most cultivated varieties need caprification or pollination by the insect *Blastophaga psenes* L. [14].

## 2. Materials and Methods

### 2.1. Vegetal Material

To the knowledge of the authors, few works differentiate between brebas and figs. Being the breba fruits, generally poor studied, characterized and almont no compared between the different varieties. Based on this, for the present study, four biferous varieties of fig trees (*Ficus carica* L.) with consolidated commercial interest were selected for fresh consumption, these being [8]:

- ‘Cuello de Dama Negro’: Biferous variety with an important breba production and very well adapted to rainfed conditions. Breba fruits present a good aptitude for manipulation and transport, fulfilling the demands of the market for their fresh consumption.
- ‘San Antonio’: Biferous variety perfectly adapted to rainfed cultivation conditions, it is interesting for the early ripening of its fruits.
- ‘Colar’: The most widespread variety in the southeast of Spain, due to its productivity and earliness, as well as its size, color, and flavor.
- SuperFig1’ (‘SF1’): Little-known parthenocarpic biferous variety, presents a productive and precocious tree. It has resistant skin and its taste quality is high.

The 15-year-old trees are located on a commercial plot in Ojós (Murcia), in southeastern Spain. The cultivation and management conditions remained homogeneous for the four varieties studied in order to reduce the environmental impact on the parameters studied. The trees are disposed of in a 6 × 4 frame and irrigated by a drip irrigation system. Each fig tree has available 4 drippers of 4 L h^−1^, with total annual consumption of water around 3500 m^3^ per ha. The fertilization was carried out between the months of March and September, using 150 functional units (UF) of nitrogen, 80 UF of P_2_O_5_, and 150 UF of K_2_O. At the time of fruit collection, all the trees presented a good phytosanitary status.

For each variety, a total of 20 brebas were collected from four different trees (5 fruits per tree). The number of figs per variety (*n* = 20) was determined according to the minimum sample size, guaranteeing the representativeness of the population with an interval of 95%, calculated by Equation (1) [15].

(1) $$n\;\geq \frac{1.96\;x\;S}{\frac{\hat{\gamma }}{10}}$$

where *n* represents the minimum representative sample size of the population; S is the standard deviation of the sample; $\hat{\gamma }$ the sample mean.

All the figs were collected manually in their commercial maturity cap, which was determined by the softening of the fruit, typical coloration according to the variety, and longitudinal cracking of the skin. Once collected, the figs were immediately transferred to the laboratory and processed and analyzed on the same day.

### 2.2. Morphological Characterization

**Figure 1:**
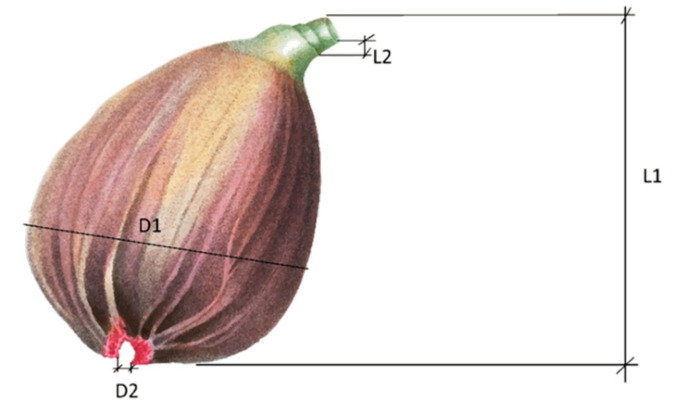
Representation of the measures considered to determine the size of the figs, where D1 represents the maximum equatorial diameter of the fig (mm); D2—diameter of the free ostiole (mm); L1—longitudinal height of the fig with peduncle (mm); L2—longitudinal height of the peduncle on its shortest side (mm).

The morphological characterization of the figs was carried out using the parameters of weight, size, and color. The weight of the figs was determined using an analytical balance (model AG204 scale; Mettler Toledo, Barcelona, Spain) with a precision of ±0.01 g, for all the fruit samples the total fruit weight and the peel weight was determined, both weights in grams. The breba size was determined with the measurements relative to the maximum equatorial diameter of the fruit (D1, mm), the diameter of the free ostiole (D2, mm), the longitudinal height of the breba with peduncle (L1, mm), and longitudinal height of the peduncle on its shortest side (L2, mm), as shown in Figure 1, all measurements were made by means of a digital Vernier. with a precision of ±0.01 mm (model 500-197-20, 150 mm; Mitutoyo Corp., Aurora, IL, USA).

The external and internal color of the brebas was established using a spectrophotometer (model CM-700d, Minolta, Osaka, Japan) according to the Commission Internationale de l’Éclairage (CIE) and was expressed as color values L *, a *, and b *; where L * indicates the lightness of the color (L * = 0 and L * = 100 represent black and white, respectively), a * it is position between green and red (negative and positive values of a * indicate green and red, respectively) and b * its position between blue and yellow (negative and positive values of b * point towards blue and yellow, respectively). The target color $\left[C^{*}=\sqrt{(a^{*2}+b^{*2}})\right]$ and Hue angle ($H^{\circ }=arctan\frac{b^{*}}{a^{*}}$) were also determined. All measurements were carried out at constant room temperature (23 ± 3 °C). The results are presented as the mean of the values (*n* = 20) and their standard deviation.

### 2.3. Chemical Characterization of Brebas

The biochemical analyzes of each breba variety were carried out on four different juice samples (*n* = 4) per variety. The juice samples were obtained from the processing of sub-samples composed of five breba fruits each, using a domestic press blender (Orbegozo LI model 5060). The juices were placed in sterile polyethene containers until their use to ensure their preservation, and TSS, TA, and pH analyses were determined the same day.

Total soluble solids (TSS, °Brix) were measured using a refractometer (PCE-0100, PCE Instruments, Alicante. Spain). The pH and titratable acidity (TA, g citric acid 100 mL^−1^) were determined using an automatic potentiometric titrator (877 Titrino plus, Metrohm). In addition, the maturity index (MI) was also calculated as the TSS/TA ratio. The results represent the mean values (*n* = 4) with their standard deviation.

### 2.4. Sugar and Organic Acid Content

For the sugars and organic acids quantification, aliquots of breba juice, 10 mL, (Sigma 3–18 K, Osterode & Harz, Germany) were previously centrifuged at 15,000 rpm for 10 min at 4 °C, then the supernatant was removed carefully and filtered with a 0.45 µm nylon membrane (Filter-Lab NY 0.45 µm). The determination of sugars and organic acids was performed by Agilent 1100 high-performance liquid chromatography (HPLC) with ChemStation software using a Supelcogel C610H column, 30 cm × 7.8 mm, and a Supelguard guard, 5 cm × 4.6 mm (Supelco, Bellefonte, PA). To measure the absorbance of fatty acids, a diode array detector (DAD) (Diode Array DAD G1315A) set at 210 nm and a refractive index detector (RID) (G-1362-A) were used for the sugar content. In both cases, reference standards of organic acids and sugars were used supplied by Sigma-Aldrich (Poole, Dorset, United Kingdom) with calibration curves with R^2^ ≥ 0.999. The results obtained are expressed in g 100 mL^−1^.

### 2.5. Determination of the Polyphenolic Profile

The polyphenolic profile of figs was identified by HPLC-DAD-ESI-MSn (Agilent 1200 series). For extraction, 50 mg of lyophilizate pulp were weighed and dissolved in 1 mL of MeOH:H_2_O:formic acid (75:24:1) using a vortex for 5 min. Subsequently, they were centrifuged for 10 min at 12000 rpm and filtered by 0.45 µm. The relative quantification of the phenolic compounds present in the samples was carried out through chromatography comparison with pure standards (chlorogenic acid, rutin, and cyanidin-3-glucoside, Sigma-Aldrich) and their maximum absorbance spectrum at an emitted wavelength. at 290 nm, 320 nm, and 520 nm, respectively, through a diode UV detector (DAD) integrated into the HPLC and connected online to the mass spectrometer.

### 2.6. Amino Acids Identification

For amino acids identification and quantification, aliquots of 50 mg of lyophilized fig pulp in a methanol/water solution (50/50 *v*/*v*) were used. The extract was vortexed for 1 min, sonicated for 3 min and finally centrifuged at 5000 rpm for 20 min at 4°C The supernatant was collected and dried in a vacuum oven at 27 °C and resuspended in 800 μL of D2O phosphate buffer (100 mM KH2PO4 pH = 6.0) containing 0.58 mM TSP (trimethylsilylpropionic acid sodium salt). The mixture was centrifuged at 4000 rpm for 5 min at 4 °C and the supernatant (600 µL) was placed in a 5 mm nuclear magnetic resonance (NMR) tube for analysis. All spectra were recorded on a Bruker AV-HD NMR operating at a proton NMR frequency of 500.16 MHz. Each 1H NMR spectrum consisted of 64 scans with the following parameters: 0.191 Hz/point, pulse width = 4, 0 μs (90°) and relaxation delay = 2.0 s. The free induction decay was Fourier transform with line broadening = 1 Hz, Gaussian broadening = 0, and sensitivity to peak selection = 1.0. 1H-NMR spectra were analyzed with Chenomx Profiler (v. 8.0., Edmonton, Canada) to obtain amino acid concentrations in figs.

### 2.7. Identification of Volatile Aromatic Compounds

Volatile compounds were identified by a Shimadzu GC-17A gas chromatographer coupled to a Shimadzu QP-5050A mass spectrometry detector (Shimadzu Corporation, Kyoto, Japan). The GC-MS system was equipped with a 30 m × 0.25 mm Supelco (Supelco, Inc., Bellefonte, PA, USA) SLB-5 MS (fused silica) column with a 0.25 µm film thickness. The carrier gas used for this analysis was helium maintained at a column flow rate of 0.6 mL min^−1^ and a total flow of 181.2 mL min^−1^ in a division ratio of 1:300. The ramp used was: 3 °C min^−1^ from 80 to 170 °C, and 25 °C min^−1^ at 300 °C, maintaining this final temperature for 1 min. The detector temperature was 300 °C and 230 °C for the injector.

### 2.8. Statistical Analysis

The significant differences of the experimental data obtained were evaluated using a one-way analysis of variance (ANOVA) followed by a contrast of means separation using the Tukey test for *p* < 0.05. In addition, for all results, principal component analysis (PCA) and cluster analysis (CiA) were also performed. Cluster analysis was applied to the standardized data for hierarchical associations employing Ward’s method for agglomeration and the squared Euclidean distance as the dissimilarity measure. Statistical analyzes were performed with the Statgraphics Centurion 18 Software data analysis software.

## 3. Results

### 3.1. Morphological Characterization

The morphological characterization results of the four fig varieties studied are presented in Table 1. It should be noted that most of the parameters studied were significant at 95% confidence level according to ANOVA, with the exception of the Hue angle (H°) for the external color and the L * coordinate for the internal color, which were not different.

The mean weight of the brebas studied ranged between 62.58–134.63 for the whole fruit and between 15.02–35.77 g for the peel. While the varieties ‘Colar’ and ‘SF1’ presented the highest weights, both of breba (133.92 g ‘SF1’) and peel (34.49 g ‘Colar’), the weights of the varieties ‘Cuello de Dama Negro’ and ‘San Antonio’ were significantly different, with values between 62.58–75.68 g and 15.02–16.07 g for whole fruit and peel, respectively. The weight results for the ‘Colar’ and ‘SF1’ varieties were only different when compared to the weights of the ‘Cuello de Dama Negro’ and ‘San Antonio’ varieties, but, in both cases, without statistical differences between them. On the other hand, the highest pulp yield was presented by the ‘Cuello de Dama Negro’ variety with an average yield of 80%. This pulp yield was significantly higher than that of the other varieties, which ranged between 73 and 74% (‘Colar’> ‘San Antonio’ = ‘SF1’).

The external and internal color of the brebas showed little variability between the studied varieties (*p* > 0.05). The luminosity parameter (L *) did not present significant differences both for the external color (with values between 25.41 and 30.05), and for the internal color (range between 51.29 and 55.65), in both cases, the results indicated luminous colors (values elevated L * and positive).

The Hue angle (H°), or objective color, presented values between 31.93 and 40.52, without significant differences between the varieties for the external color, while for the internal color, the highest tone angle (81.00) was determined for the ‘San Antonio’ variety. This result is significantly higher when compared to the other varieties, which ranged from 54.45 to 58.40.

Regarding the external color, the variety ‘Cuello de Dama Negro’ exhibited values for the parameters a* (2.51) and b* (1.39) well below the other varieties, which remained without significant differences in the ranges of 7.32–8.69 and 5.68–7.44 for parameters a* and b*, respectively. For the internal color, the varieties ordered from highest to lowest a* were established as: ‘Cuello de Dama Negro’ > ‘Colar’ > ‘SF1’ > ‘San Antonio’, highlighting the difference between the maximum result (14.36 for ‘Cuello de Dama Negro’) and the lowest value obtained (3.41) for ‘San Antonio’. Based on the results obtained for the internal color parameter b*, the varieties can be divided into two groups, on the one hand, ‘Cuello de Dama Negro’ and ‘San Antonio’, which presented the highest values (19.86 and 21.43, respectively), and on the other, the varieties’ SF1’ and ‘Colar’, with values > 17 and >18.

### 3.2. Chemical Characterization of Brebas

In relation to the chemical characterization of breba fruits, the four varieties presented pH values between 8.35 and 8.59, without significant differences between them. The ‘Cuello de Dama Negro’ variety (17.25 °Brix) showed the highest levels of total soluble solids, while the lowest amount was determined for the ‘Colar’ variety (12.37 ° Brix). On the other hand, the variety ‘Colar’ was the one that presented the highest TA value (1.43 g citric acid L^−1^) significantly different when compared to the TA of the varieties that presented the lowest values, these being ‘Cuello de Dama Negro’ and ‘San Antonio’ with 1.14 and 1.18 g citric acid L^−1^, respectively. The MI for all varieties was between 129.62 and 153.94, but without statistically significant differences. The results are presented in Table 2.

### 3.3. Sugar and Organic Acid Content

The sugars and organic acids present in the breba varieties studied are shown in Table 3. In general, citric and malic acids were identified in all varieties of figs, constituting the main organic acids. The varieties ‘San Antonio’, ‘SF1’ and ‘Colar’ presented a higher concentration of malic acid than citric acid, while for the variety ‘Cuello de Dama Negro’, the amount of citric acid was greater than that of malic acid (4.89 g kg^−1^ and 3.24 g kg^−1^, respectively). Succinic acid was detected between 0.222–0.267 g kg^−1^ in all varieties (‘San Antonio’ > ‘Colar’ > ‘Cuello de Dama Negro’) except for the breba ‘SF1’, in which it was not detected.

For the ‘San Antonio’ breba was identified the highest content of formic acid, with a concentration of 0.119 g kg^−1^, while, for the other three varieties, the acid content was less than 0.1 g kg^−1^, without differences significant among them. Lactic acid was also detected in the four varieties, where the ‘Colar’ variety presented the highest amounts (0.292 g kg^−1^) and ‘Cuello de Dama Negro’ the lowest values (0.153 g kg^−1^), the results for all the varieties showed significant differences.

In general, the variety ‘SF1’ presented the highest content of organic acids (11.584 g kg^−1^), followed by ‘Colar’ (10.444 g kg^−1^) > ‘San Antonio’ (9.506 g kg^−1^) > ‘Cuello de Dama Negro’ (8.630 g kg^−1^).

In relation to the sugars contained in the breba juices, fructose and glucose were the major sugars in all the juices studied. In this sense, the variety ‘Cuello de Dama Negro’ presented the highest concentrations of glucose (131.187 g kg^−1^), and ‘San Antonio’ of fructose (122.66 g kg^−1^), while the lowest results, for both sugars, were determined for the variety ‘SF1’, with 100.07 g kg^−1^ of fructose and 99.59 g kg^−1^ of glucose. Sucrose was also detected in all samples, with maximum concentrations between 4.6 and 4.8 g kg^−1^ for the ‘Cuello de Dama Negro’ and ‘San Antonio’ varieties, respectively. On the contrary, the lowest values of sucrose were determined for ‘Strain’ (3.1 g kg^−1^) and ‘SF1’ (3.5 g kg^−1^).

‘Cuello de Dama Negro’ variety presented the highest total sugar content (253.73 g kg^−1^) followed by the varieties ‘San Antonio’ (248.30 g kg^−1^) > ‘Colar’ (208.43 g kg^−1^) > ‘SF1’ (203.24 g kg^−1^).

The differences in the total content of both sugars and organic acids in the juice of the different breba varieties is clearly seen in the significant differences in the sugar/organic acid ratio between the varieties, where ‘Cuello da Dama Negro’ presented the higher ratio (29.4) and ‘SF1’ (17.5) the lower.

### 3.4. Determination of the Polyphenolic Profile

Due to the important influence on the fruit quality, mainly caused by its impact on parameters such as flavor and odor, the phenolic profile of breba fruits was determined by identifying and quantifying the polyphenolic compounds as shown in Table 4. Three of the four identified polyphenols (caffeic acid 4-O, 5-CQA, and cyanidin 3-rutinoside) were detected in the four varieties.

‘Cuello de Dama Negro’ variety stood out for showing the highest contents for all compounds compared to the other varieties. In this sense, while the concentrations for the cyanidin 3-rutinoside, caffeic acid 4-O, and 5-CQA compounds in ‘Cuello de Dama Negro’ breba’s were 1.897 mg g^−1^, 1.231 mg g^−1^ and 0.278 mg g^−1^ respectively, in the other varieties, the concentrations detected were at least 30% lower.

In all cases, ‘Colar’ variety presented the lowest values, with contents of 0.444 mg g^−1^ for Caffeic acid 4-O; 0.05 mg g^−1^ for 5-CQA; and 0.210 mg g^−1^ for cyanidin 3-rutinoside. Additionally, ‘Cuello de Dama Negro’ was the only variety in which quercetin 3-(6-O-acetyl-beta-glucoside) was detected with a concentration of 0.153 mg g^−1^. The heterogeneity of the results, with distant maximums and minimums, is confirmed with the statistical study since all the results showed significant differences between the varieties.

### 3.5. Amino Acids and other Metabolites

In order to study and identify in detail the breba fruits and their possible compositional variations related to the variety, a metabolomic characterization was carried out due to their metabolic and nutritional importance (Table 5).

A total of eleven amino acids were identified, among them, only aspartate and valine did not show significant differences between the varieties. The brebas of the varieties ‘Cuello de Dama Negro’ and ‘San Antonio’ showed the highest glutamate and glutamine contents, with concentrations between 0.539–0.604 mM and 0.813–0.856 mM, respectively, while the varieties ‘SF1’ and ‘Colar’ showed values around 0.3 mM of glutamate and between 0.652 mM and 0.455 mM of glutamine, respectively.

In the ‘San Antonio’ brebas, the highest concentrations of alanine (0.759 mM) and proline (1.44 mM) were detected; meanwhile, the variety ‘SF1’ presented the highest contents of isoleucine (0.153 mM) and leucine (0.062 mM), the latter together with the variety ‘Colar’ (0.068 mM), and the lowest contents of proline (0.439 mM). On the other hand, the ‘Cuello de Dama Negro’ brebas showed the lowest concentrations of alanine (0.998 mM), isoleucine (0.052 mM), and leucine (0.041 mM). The highest GABA values were identified in the ‘San Antonio’ fruits (0.528 mM) and ‘Colar’ (0.461 mM) while ‘Cuello de Dama Negro’ (0.358 mM) and ‘SF1’ (0.378 mM) presented the lower values, without significant differences between both groups. The variety ‘Colar’ showed the highest concentrations of asparagine (12,041 mM) while the other varieties showed ≤ 8704 mM (‘Cuello de Dama Negro’) but without differences between them. The only variety in which tyrosine was detected, with a concentration of 0.018 mM.

On the other hand, two additional metabolites were identified, choline and trigonelline. Regarding choline, ‘Cuello de Dama Negro’ (0.169 mM) showed the lowest concentration compared to the other varieties, which was around 0.2 mM. Already for trigonelline, the results showed significant differences between the varieties, where for ‘Colar’ it was not detected and for the others ‘San Antonio’ > ‘Cuello de Dama Negro’ > ‘SF1’.

### 3.6. Volatile Compounds

Among the four breba varieties studied, a total of 80 different volatile compounds were identified (Table 6). For the ‘San Antonio’ brebas, 47 different volatile compounds were identified, making it the variety with the greatest diversity of volatile compounds, followed by ‘SF1’ (39) > ‘Colar’ (22) > ‘Cuello de Dama Negro’ (20).

Only seven of the eighty volatile compounds were detected in the four varieties, these being: benzyl alcohol; benzaldehyde; cycloheptasilo1ane, tetradecamethyl-; 3-ethyl-4-methylpentan-1-ol; (3R, 6S)-2,2,6-trimethyl-6-vinyltetrahydro-2H-pyran-3-ol; cyclopentasilo1ane, decamethyl-; and cyclotetrasilo1ane, octamethyl-.

### 3.7. Principal Component Analysis (PCA)

Aiming to better understand the trends and relationships between the many variables studied (52) for the different samples of brebas (four varieties), principal component analysis (PCA) was applied. The first three principal components (PC) explained 100% of the total variation. The first two PCs explained 91.04% of the observed variability. The first component (PC1), which represents 50.61% of the total variance, was related to the analyzed variables trigonelline, sucrose, fructose, glucose, fruit weight, peel weight, D1, D2, L1. L* external, L* internal, b* internal, AT, pH, SST, glutamate, glutamine, isoleucine, and leucine. These variables are related to the most important sugars, fruit size, exterior and interior brightness of the fruit, acidity, and TSS, as well as various amino acids.

**Figure 2:**
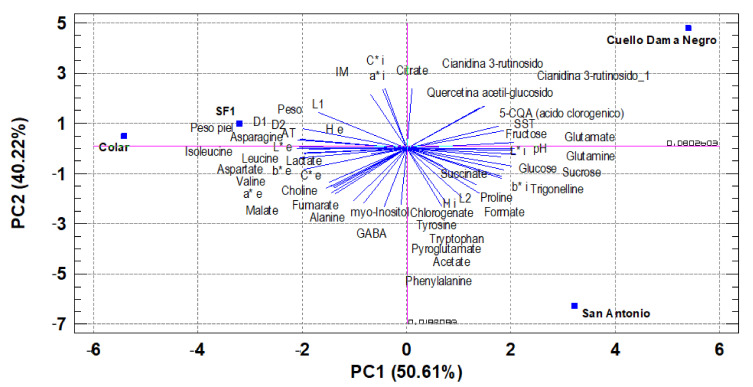
Principal component analysis (PC1-PC2) of morphological and chemical parameters.

PC2 represented 40.42% of the total variance. It was correlated with the content of Choline, Chlorogenate, myo-Inositol, L2 a* external, C* external, a* internal, H° internal, C* internal, MI (TSS/TA), quercetin acetyl-glucoside, cyanidin 3-rutinosido_1, GABA, alanine, aspartate, phenylalanine, pyroglutamate, tryptophan, and tyrosine (Figure 2).

**Figure 3:**
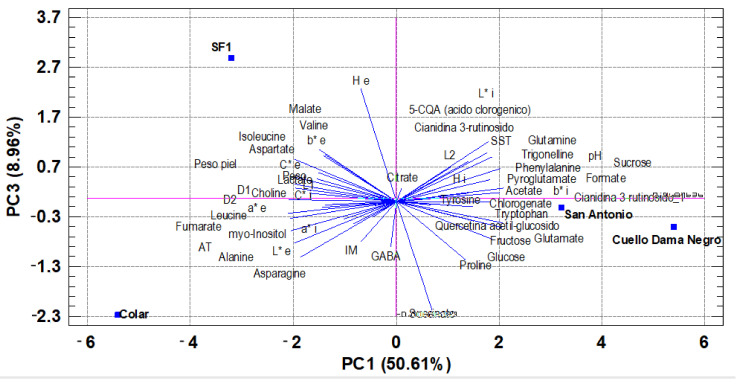
Principal component analysis (PC1-PC3) of morphological and chemical parameters.

PC3 represented 8.96% of the observed variability and was correlated with succinate, b* external, H° external, 5-CQA (chlorogenic acid), quercetin acetyl-glucoside, and proline. (Figure 3).

The PCA results showed that the PC1 axis allows clearly discriminating the ‘San Antonio’ and ‘Cuello de Dama Negro’ varieties from the ‘Colar’ and ‘SF1’ cultivars. (Figure 2). PC2 allows discriminating the ‘San Antonio’ cultivar from the other three.

**Figure 4:**
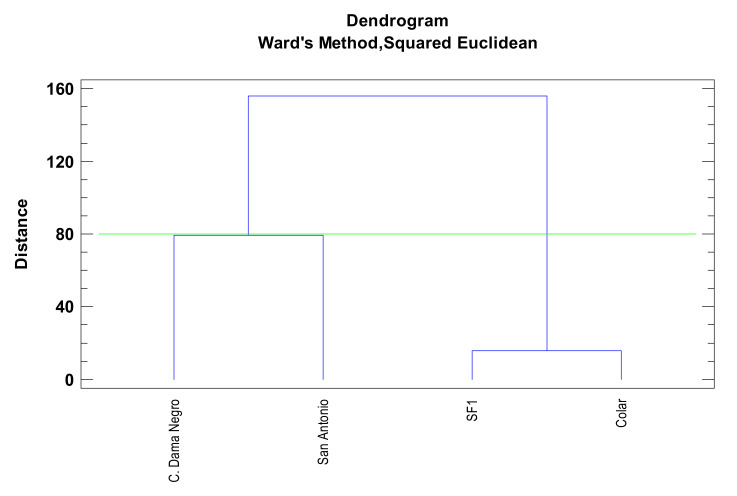
Dendrogram of the Ficus carica L varieties using Ward’s method based on squared Euclidean distance from morphological and chemical parameters.

PC3 helps to discriminate the ‘Colar’ cultivar from the other three and the SF1 cultivar from the other three. The morphological and chemical parameters dendrogram showed and confirmed the differences between the brebas varieties studied but indicated similarities between ‘Colar’ and ‘SF1’ varieties and ‘Cuello de Dama Negro’ and ‘San Antonio’ cultivars (Figure 4).

## 4. Discussion

Regarding the morphological characteristics, it should be noted that, for all the varieties considered in the present work, the fruit weight and size were higher than that indicated by other authors. In this sense, while the lowest and highest fruit weight of the studied varieties was 62.58 g (‘San Antonio’) and 134.63 g (‘Colar’), respectively, in a study carried out by Çalişkan and Polat [11] for two years with more than eight Tunisian varieties, the maximum and minimum fruit weight recorded was 52.5 g (31-IN-17) and 22.2 g (Ufak Yesil), respectively. This weight difference is also related to the fruit size, the Turkish ones being considerably smaller (with fruit length between 44.2 mm and 31.9 mm) than those studied here (between 74.25 mm and 96.81 mm) [11]. The same trend regarding larger size and weight is observed when compared with different varieties of figs such as Turkish, Lebanese, and Indian [16,17,18]. However, fewer differences were identified when comparing the results with those indicated for other Spanish varieties [19]. In this study, Pereira et al. [19] characterized seven Spanish varieties grown in southwestern Spain, and despite also working with the ‘Colar’ and ‘San Antonio’ varieties, both the weights (47.6 g for ‘San Antonio’ and 76.5 g for ‘San Antonio’), as the length of the fruit (55.8 mm ‘San Antonio’ and 74.7 mm ‘Colar’) were shorter. This may be due to a greater influence of edaphoclimatic conditions compared to genetics [20].

Considering the color diversity presented by the different fig varieties worldwide, and which can vary from green/yellowish tones to intense blacks, in general, consumers common trend, showing a preference for figs that have dark colors and intense for fresh consumption [5]. In this sense, this study focused on the identification, characterization, and quantification of the morphological and chemical-nutritional properties of the four most relevant varieties of figs in the southeast of Spain, from the economic and commercial point of view. Thus, the results obtained for the external color were consistent with those reported by Anat et al. [21] for ‘Bursa’ (L* = 25.3; a* = 8.8; b * = 3.3; C = 9.4; H* = 20.7) and ‘Chechick’ (L* = 27.5; a* = 2.3; b* = 1.3; C = 1.8; H* = 29.5). Despite these similarities, it should be noted that the breba fruits present a faster maturity period when compared with the figs, and in cooler temperatures. These temperatures and maturity time differences can explain the diversity of color, but also the chemical composition of the fruits [22].

The analysis of TSS, TA, and MI (TSS/TA) parameters are important in the evaluation of the quality of the fruit, and are used to define its levels of maturity, and, therefore, its quality attributes. This means that high TSS and MI (TSS/TA) values will imply greater acceptance by the consumer [1]. In this sense, the TSS values for the four varieties were higher than those indicated in Turkish (11 °Brix) and Indian (9 °Brix) varieties [23,24], however, are inferior when compared to the same varieties. Souza et al. [25] indicated values of 18.43 °Brix for ‘Colar’, 17.40 °Brix for ‘San Antonio’ and 17.00 °Brix for ‘Cuello de Dama Negro’, this may be due to the fact that the authors characterized the figs and not the breba fruits, being, generally, the figs much sweeter than the figs [8]. Although the ideal MI (TSS/TA) may vary in relation to the final use/consumption of the fruit, varieties with high MI (TSS/TA) values are generally preferred. The MI (TSS/TA) values obtained between 129.62 and 153.94 indicate the commercial suitability for consumption of the four varieties studied at the time of harvest, confirming their commercial maturity. Similar values for Spanish varieties were indicated by Pereira et al. [19,26]. However, these values are higher when compared to the Turkish varieties ‘Bardak’ and ‘Dolap’, which presented a MI (TSS/TA) mean value around 79.8 [11]. Note the difficulty to compare the results with other research, since in most of the published works the fig fruit type (fig or breba) is not defined or determined.

Brebas, such as figs, represent a role in the Mediterranean diet, with frequent consumption in its various forms (fresh, dry, and/or processed). Their high appreciation and consumption are due to their fluffy, fresh, and sweet texture [27]. For the four varieties studied, glucose and fructose were confirmed as the main sugars in brebas, which agree with the literature [2,28,29]. Although, in the 1990s, some authors indicated that sucrose would not be present in breba juices [30], most studies, such as this one, have shown that this statement is not correct, once the identification of this sugar in fig fruits becomes common [3,31].

Since both the amount and the composition of the sugars have a direct influence on the sweetness of the fruit and its taste appreciation, the results indicate differences between the varieties, since for ‘Cuello de Dama Negro’ and ‘SF1’ their sweetness is mainly due to fructose, while for ‘San Antonio’ and ‘Colar’ it would be glucose. The values obtained are higher than those obtained in other studies [11,26,32]. This difference can be explained by the methodology used since the characterization of the sugars in this study was carried out with lyophilized material, that is, dry weight, while in the others it is reported as fresh weight. It is important to note that the determination in dry weight (lyophilized) usually results in higher concentrations (due to the concentration of the compounds due to the elimination of water) compared to the determinations on fresh weight.

However, the sweetness of the fruit is not only related to sugars, but also to organic acids. Most studies indicate malic and citric acid as the main organic acids of figs [32], which confirms the results obtained in the present work for the four varieties studied. The citric acid contents of the ‘Cuello de Dama Negro’ and ‘SF1’ varieties were similar to those identified for the Miljska Figa variety (4.6 g kg^−1^), while the Crna petrovka varieties (3.95 g kg^−1^) and Bela petrovka (2.7 g kg^−1^) for ‘Colar’ and Santo Antonio, respectively [33]. However, in the same study, Veberic et al. [33] determined for the Slovak varieties much lower amounts of malic acid (1.06–2.17 g kg^−1^) compared to those determined in the present work. The higher content of organic acids can result in a less sweet appreciation in the flavor of the fruit, counteracting the sweetness provided by the sugars. As far as the authors are aware, no work has been reported where the presence of lactic acid in brebas is reported.

On the other hand, with their use and consumption increasingly widespread throughout the world, brebas have shown to be an excellent source of phenolic compounds [34]. All the identified compounds are consistent with the literature [2,20,31]. Despite the great differences in the 5-CQA content between the varieties, the results are shown in agreement with the literature. Oliveira et al. [35] already reported 5-CQA concentrations between 0.329 mg g^−1^ and 0.028 mg g^−1^ for the Portuguese varieties ‘Pingo de Mel’ and ‘Branca Tradicional’. It should be noted that, in the same study, the authors did not detect the flavonoid glycoside, quercetin 3-glucoside, in the fruits, only in the peel and leaves, while, in the present study, it was identified in ‘Cuello de Dama Negro’. This fact is not unusual, since protective effects of cell structures against UV radiation are attributed to this compound, as well as a high influence on the coloring of the fruits, where ‘Cuello de Dama Negro’ presented a pulp with a much more intense coloration [36,37]. Cyanidin 3-rutinoside is mainly attributed to antioxidant functions, defense against pathogens, and pigmentation [38,39], so its higher content in ‘Cuello de Dama Negro’, in comparison with the other varieties, would again be related to its specific intense coloration. The results were in line with those reported by Wojdyło et al. [31] for the varieties ‘Colar’, ‘San Antonio’, and ‘Cuello de Dama Negra’ grown under similar conditions.

Amino acids are building blocks of proteins and polypeptides. In addition to the nutritional and clinical properties, some amino acids can influence the perception of taste, since they can present a sweet taste [40]. To the authors knowledge, there are no references on the identification and quantification of amino acids in figs and their role in specific plant metabolism, which highlights the contribution of this work. In general terms, the great variety of amino acids identified would indicate the great benefits and potentialities for human health that the consumption of figs presents. In this context, Pasiakos et al. [41] showed that supplementary amounts of branched-chain amino acids such as leucine, isoleucine, and valine, accelerate the recovery of muscle damage, pain, and fatigue after exercise, therefore, the varieties ‘SF1’ and ‘Colar’ would be the most appropriate, at a time they show higher amounts of those amino acids. It is striking that tyrosine was only detected for ‘SF1′, indicating the high biological potential of the variety, since Neyra et al. [42] associated tyrosine with crucial physiological events, its oxidation being able to cause beneficial or harmful effects on the biological systems. Other amino acids detected in fruits, such as proline, glutamine, and asparagine, are directly related to cancer therapy [43].

On the other hand, in addition to the mentioned characteristics, the quality of the fruits is also affected by other parameters, such as aroma. Several studies indicate that volatile compounds significantly affect flavor and aroma quality [44]. Despite the fact that the studies focused on the volatile compounds of figs are limited since in most of them they are detected in the leaves and not in the fruits. The results were shown to be in line with those supplied for Portuguese varieties of figs [45], however, the diversity and variability of volatile compounds can be related to the diversity of metabolic routes from which they can be derived, since they can come from amino acids and organic acids [46].

## 5. Conclusions

Based on the experimental results, the following points can be concluded:

‘Cuello de Dama Negro’ was the variety with the highest amount of TSS (17.25 °Brix), with significant differences over ‘San Antonio’ and ‘SF1’, and these in turn also present significant differences over ‘Colar’ (12.37 °Brix). However, there are no differences between the four varieties regarding MI.

Malic and citric acids were the predominant organic acids in breba fruits. Malic acid predominates significantly in ‘San Antonio’, ‘SF1’ and ‘Colar’ while ‘Cuello de Dama Negro’ has a significantly lower content. Citric acid is presented in higher quantity in ‘Cuello de Dama Negro’ compared to ‘San Antonio’.

The variety with the greatest diversity and content of phenolic compounds was ‘Cuello de Dama Negro’. The phenolic compound cyanidin 3-rutinoside was only detected in the ‘Cuello de Dama Negro’ fruits.

The amino acid tyrosine was only detected in ‘SF1’, while the metabolite trigonelline was presented in all varieties except ‘Colar’.

The varieties presented a great diversity of volatile compounds, where only seven of the eighty compounds were detected in all the varieties. Further studies need to be carried out to verify the potential of establishing a form of varietal identification based on the presence and/or absence of volatile compounds.

This is the first study that compares and characterizes the chemo-nutritional and pomological properties of the four varieties of figs that are most relevant from the commercial point of view of southeastern Spain.

Due to its content in bioactive compounds, the consumption of this fruit can contribute to the prevention of certain diseases, however, more specific studies are needed to corroborate this statement.

An important knowledge gap was identified in relation to the characterization of the two crops of Ficus carica L., that is, the differentiation and specification in the literature when working with brebas and/or figs.

## Figures and Tables

**Table 1:** Morphological characteristics of brebas fruits obtained from four varieties of fig trees (*Ficus carica* L.) grown under homogeneous conditions. The values presented are the mean values (*n* = 20) and their standard deviation in parentheses.

	Variety			
	‘Cuello de Dama Negro’	‘San Antonio’	‘SF1’	‘Colar’
Fruit weight (g)	75.68 (9.20) a	62.58 (8.93) a	133.92 (20.83) b	134.63 (20.75) b
Peel weight (g)	15.02 (1.99) a	16.07 (2.81) a	35.77 (5.45) b	34.49 (5.29) b
Pulp yield (%)	80 a	73 b	73 b	74 b
Fruit diameter (D1) (mm)	47.51 (1.73) a	48.16 (4.10) a	60.15 (4.14) b	63.07 (3.44) b
Ostiolo width (D2) (mm)	2.44 (0.55) a	2.64 (0.45) a	3.17 (1.13) ab	3.49 (1.07) b
Fruit length (L1) (mm)	85.54 (6.89) a	74.25 (4.19) b	95.72 (4.19) c	96.81 (6.16) c
Peduncle length (L2) (mm)	3.95 (1.27) a	6.07 (2.05) b	4.29 (1.43) a	3.32 (1.10) a
External Color				
L*	25.41 (0.65) a	26.42 (1.51) a	27.58 (3.36) ab	30.05 (3.30) b
a*	2.51 (1.27) a	8.38 (1.77) b	7.32 (2.20) b	8.69 (2.50) b
b*	1.39 (0.40) a	5.86 (1.91) b	7.44 (4.17) b	5.68 (2.41) b
Hue angle (H°) ^1^	31.94 (9.03) a	34.12 (5.75) a	40.52 (13.08) a	31.93 (6.75) a
Target color (C)	2.93 (1.26) a	10.30 (2.39) b	10.84 (4.11) b	10.57 (3.04) b
Internal Color				
L* ^1^	55.52 (3.77) a	55.65 (6.43) a	54.47 (6.69) a	51.29 (4.29) a
a*	14.36 (2.44) a	3.41 (1.01) c	11.25 (2.46) b	12.70 (3.01) ab
b*	19.86 (1.45) a	21.43 (1.88) a	17.91 (1.99) b	17.31 (1.63) b
Hue angle (H°)	54.45 (5.07) b	81.00 (2.43) a	58.40 (4.84) b	54.49 (4.49) b
Target color (C)	24.65 (1.57) a	10.30 (2.36) c	21.27 (2.78) b	21.60 (2.89) b

The different letters within the rows indicate significant differences according to the Tukey test (*p* < 0.05). ^1^ No significant differences were determined by ANOVA (*p* < 0.05).

**Table 2:** Total soluble solids (TSS), titratable acidity (TA) and maturity index (MI) of the brebas obtained from four cultivars of fig trees (*Ficus carica* L.) grown under homogeneous conditions. The values presented are the mean values (*n* = 4) and their standard deviation in parentheses.

	Variety			
Parameter	‘Cuello de Dama Negro’	‘San Antonio’	‘SF1’	‘Colar’
pH	8.59 (0.4) a	8.58 (0.4) a	8.43 (0.9) a	8.35 (0.6) a
TSS (°Brix)	17.25 (0.37) a	15.25 (0.37) b	15 (1.0) b	12.37 (0.37) c
TA (g citric acid L^−1^)	1.14 (0.08) b	1.18 (0.06) b	1.3 (0.01) ab	1.43 (0.14) a
MI (TSS/TA)	152.50 (11.36) a	129.62 (5.28) a	144.48 (24.10) a	153.94 58.73) a

The different letters within the rows indicate significant differences according to the Tukey test (*p* < 0.05).

**Table 3:** Content of organic acids (g kg^−1^) and sugars (g kg^−1^) identified in the breba juice of four varieties (*Ficus carica* L.) grown under homogeneous conditions. The results represent the mean values (*n* = 3) with their standard deviation in parentheses.

	‘Cuello de Dama Negro’	‘San Antonio’	‘SF1’	‘Colar’
Organic acids				
Citrate	4.895 (0.17) b	2.303 (1.73) a	4.108 (0.12) ab	3.671 (0.17) ab
Formate	0.081 (0.00) a	0.119 (0.00) b	0.068 (0.01) a	0.061 (0.00) a
Fumarate	0.035 (0.00) a	0.097 (0.00) c	0.065 (0.00) b	0.087 (0.00) c
Lactate	0.153 (0.004) a	0.230 (0.005) b	0.277 (0.006) c	0.292 (0.001) d
Malate	3.244 (0.10) a	6.490 (0.41) b	7.067 (0.61) b	6.073 (0.10) b
Succinate	0.222 (0.016) a	0.267 (0.008) b	ND	0.260 (0.007) b
**∑ organic acid**	**8.630**	**9.506**	**11.584**	**10.444**
Sugars				
Fructose	131.187 (0.08) b	120.780 (0.27) b	100.070 (0.27) a	101.307 (0.03) a
Glucose	117.947 (0.34) b	122.662 (1.65) b	99.592 (3.10) a	104.005 (3.31) a
Sucrose	4.603 (0.08) b	4.858 (0.27) b	3.586 (0.27) a	3.117 (0.03) a
**∑ sugar content**	**253.737**	**248.300**	**203.247**	**208.430**
*Sugar/organic acid ratio*	*29.403*	*26.119*	*17.544*	*19.957*

The different letters within the rows indicate significant differences according to the Tukey test (*p* < 0.05). ND: Not detected.

**Table 4:** Phenolic compounds (mg g^−1^) identified in the brebas fruits of four cultivars (*Ficus carica* L.) grown under homogeneous conditions. The results represent the mean values (*n* = 3) with their standard deviation in parentheses.

	Variety			
Fenolic Compound (mg g^−1^)	‘Cuello de Dama Negro’	‘San Antonio’	‘SF1’	‘Colar’
Caffeic acid 4-O	1.231 (0.019) d	0.599 (0.006) b	0.839 (0.033) c	0.444 (0.012) a
5-CQA (chlorogenic acid)	0.278 (0.005) c	0.172 (0.004) b	0.179 (0.012) b	0.050 (0.007) a
Quercetin 3-(6-O-acetyl-beta-glucoside)	0.153 (0.005) a	ND	ND	ND
Cyanidin 3-rutinoside	1.897 (0.026) d	0.313 (0.016) b	0.439 (0.022) c	0.210 (0.010) a

The different letters within the rows indicate significant differences according to the Tukey test (*p* < 0.05). ND: not detected.

**Table 5:** Concentration of amino acids and metabolites (mM) identified in four varieties of breba fruits (*Ficus carica* L.) grown under homogeneous conditions. The results represent the mean values (*n* = 3) with their standard deviation in parentheses.

	‘Cuello de Dama Negro’	‘San Antonio’	‘SF1’	‘Colar’
Amino acids (mM)				
GABA	0.358 (0.022) a	0.528 (0.043) b	0.378 (0.003) a	0.461 (0.023) b
Alanine	0.998 (0.011) d	1.759 (0.023) a	1.368 (0.081) b	1.604 (0.069) c
Asparagine	8.704 (0.046) a	9.159 (0.104) a	9.820 (0.698) a	12.041 (0.828) b
Aspartate	0.429 (0.020) a	0.518 (0.020) a	0.534 (0.108) a	0.523 (0.077) a
Glutamate	0.604 (0.047) a	0.539 (0.049) a	0.341 (0.018) b	0.339 (0.004) b
Glutamine	0.856 (0.047) a	0.813 (0.049) a	0.652 (0.008) b	0.455 (0.034) c
Isoleucine	0.052 (0.005) a	0.084 (0.002) b	0.153 (0.004) d	0.130 (0.007) c
Leucine	0.041 (0.001) a	0.049 (0.003) b	0.062 (0.001) c	0.068 (0.004) c
Proline	0.969 (0.071) b	1.411 (0.009) c	0.439 (0.002) a	0.821 (0.127) b
Tyrosine	ND	ND	0.018 (0.002) a	ND
Valine	0.100 (0.005) a	0.196 (0.107) a	0.217 (0.005) a	0.187 (0.011) a
Others metabolites (mM)				
Choline	0.169 (0.002) a	0.224 (0.012) b	0.205 (0.107) b	0.216 (0.003) b
Trigonelline	0.022 (0.000) a	0.029 (0.001) c	0.013 (0.002) b	ND

The different letters within the rows indicate significant differences according to the Tukey test (*p* < 0.05). ND: not detected.

**Table 6:** Volatile compounds identified in the four breba varieties (*Ficus carica* L.) grown under homogeneous conditions. Results (x) indicate the presence of the compound.

	Variety			
Volatile Compound	‘Cuello de Dama Negro’	‘San Antonio’	‘SF1’	‘Colar’
2,5-Furandione, dihydro-3-methylene-		X		
Silikonfett			X	
2,7-Anhydro-l-galacto-heptulofuranose		X		
3-Deoxy-d-mannoic lactone		X		
1,4-Diacetyl-3-acetoxymethyl-2,5-methylene-l-rhamnitol		X		
Oxime-, methoxy-phenyl-_			X	X
Nonanediamide, N,N’-di-benzoyloxy-	X			
Carbonic acid, hexyl methyl ester			X	
Benzyl alcohol	X	X	X	X
Benzaldehyde	X	X	X	X
2,4-Dihydroxy-2,5-dimethyl-3(2H)-furan-3-one		X		
2-Propenal, 3-phenyl-		X		
1,2,3-Propanetriol, 1-acetate		X		
Silanol, trimethyl-			X	
Silanediol, dimethyl-				X
4H-Pyran-4-one, 3,5-dihydroxy-2-methyl-		X		
Disiloxane, hexamethyl-				X
Cycloheptasiloxane, tetradecamethyl-	X	X	X	X
Hexasiloxane, tetradecamethyl-			X	
Pyridine			X	
1-Hexanol			X	X
2-Butenal, 2-methyl-		X		X
Heptanal			X	
1-Octanol			X	
Hexadecanoic acid, methyl ester		X	X	X
Decane	X			X
Nonanal	X	X	X	
Dimethylamine	X	X	X	
1,3-Propanediol, 2-(hydroxymethyl)-2-nitro-		X		
2H-Pyran-3-ol, 6-ethenyltetrahydro-2,2,6-trimethyl-			X	
Acetic acid, hexyl ester		X		
2,2-Dimethylpropanoic anhydride		X		
cis-.alpha.-Bergamotene			X	
(S)-(+)-2-Amino-3-methyl-1-butanol		X		
Valeric anhydride		X		
4H-Pyran-4-one, 2,3-dihydro-3,5-dihydroxy-6-methyl-		X		
1,2-Cyclopentanedione		X		
Dodecane, 2,6,11-trimethyl-			X	
Furaneol		X		
Azulene, 1,2,3,5,6,7,8,8a-octahydro-1,4-dimethyl-7-(1-methylethenyl)-, [1S-(1.alpha.,7.alpha.,8a.beta.)]-			X	X
1,3-Dioxol-2-one,4,5-dimethyl-		X		
3-Ethyl-4-methylpentan-1-ol	X	X	X	X
(3R,6S)-2,2,6-Trimethyl-6-vinyltetrahydro-2H-pyran-3-ol	X	X	X	X
(2-Aziridinylethyl)amine				X
2-Buten-1-ol, 2-methyl-			X	
Benzaldehyde, 4-ethyl-			X	
Ethyl propionylacetate		X		
Acetoin	X			
Cyclohexasiloxane, dodecamethyl-	X	X	X	
Heptasiloxane, hexadecamethyl-			X	
Cyclopentasiloxane, decamethyl-	X	X	X	X
Cyclotrisiloxane, hexamethyl-	X		X	X
2(3H)-Furanone, dihydro-4-hydroxy-		X		
Arsenous acid, tris(trimethylsilyl) ester			X	
Cyclotetrasiloxane, octamethyl-	X	X	X	X
Cyclooctasiloxane, hexadecamethyl-		X	X	
Cyclononasiloxane, octadecamethyl-			X	
n-Hexadecanoic acid		X		
1,7-Octadien-3-ol, 3,7-dimethyl-			X	
Phenylethyl Alcohol		X	X	
2-Furancarboxaldehyde, 5-methyl-		X		
Hexadecanoic acid, ethyl ester		X		X
Tetradecane	X		X	X
Thymine		X		
Hexanal	X			
2-Cyclohexene-1-methanol, 2,6,6-trimethyl-	X	X	X	
2-Hexenal, (E)-			X	
5-Hydroxymethylfurfural		X		
Carbamodithioic acid, diethyl-, methyl ester			X	
Butanoic acid, 2-methyl-, ethyl ester	X			X
1,2-Oxaborolane, 2-ethyl-4,5-dimethyl-	X			
1H-Indene, 1-methyl-		X		
Linalool		X	X	
Acetic acid, methyl ester		X		
3-Amino-2-oxazolidinone		X		
Dibutyl phthalate		X	X	X
Methane, nitroso-				X
4-Cyclopentene-1,3-dione		X		
Benzoic acid, methyl ester	X			
2-Furanmethanol		X		
Furfural		X		

## Data Availability

Not applicable.

## Abbreviations

SF1:	SuperFig 1 breba
D1:	Maximum equatorial diameter
D2:	Diameter of the free ostiole
L1:	Longitudinal height of the with peduncle
L2:	Longitudinal height of the peduncle on its shortest side
C*:	Target color
H°:	Hue angle
TSS:	Total soluble solids
TA:	Titratable acidity
MI:	Maturity index
HPLC:	High-performance liquid chromatography
DAD:	Diode array detector
NMR:	Nuclear magnetic resonance
ANOVA:	Analysis of variance
PCA:	Principal component analysis
CiA:	Cluster analysis
